# Minimally invasive orthodontic management: a paradigm shift in interceptive care. Part II - early management of oral habit-related malocclusions

**DOI:** 10.1590/2177-6709.31.4.e26spe4

**Published:** 2026-10-12

**Authors:** Ana Cláudia de Castro Ferreira CONTI, Felicia MIRANDA, Daniela GARIB, Amanda LOPES, Cibele ALBERGARIA, Silvio Augusto BELLINI-PEREIRA, Camila MASSARO

**Affiliations:** 1 Universidade de São Paulo, Faculdade de Odontologia de Bauru, Department of Orthodontics (Bauru/SP, Brazil).; 2 Universidade de São Paulo, Hospital de Reabilitação de Anomalias Craniofaciais, Division of Dentistry (Bauru/SP, Brazil).; 3 Private Practice (Brasília/DF, Brazil).

**Keywords:** Interceptive Orthodontics, Malocclusion, Open bite, Ortodontia interceptora, Má oclusão, Mordida aberta

## Abstract

**Introduction:**

Deleterious oral habits are common during childhood and, when persistent, may influence craniofacial growth and occlusal development. Among the malocclusions associated with oral habits, anterior open bite (AOB) and posterior crossbite (PCB) are particularly noteworthy.

**Objective:**

This article presents a conceptual and clinical overview of oral habits and their impact on occlusal development, with emphasis on early diagnosis and Minimally Invasive Orthodontic (MIO) management.

**Results:**

The literature describes associations between non-nutritive sucking, tongue interposition, mouth breathing, and occlusal alterations, especially dentoalveolar AOB and transverse maxillary deficiency. The reported interceptive approaches focus on habit interruption, combined with simple orthodontic appliances and interdisciplinary management.

**Conclusions:**

An interdisciplinary orthodontic approach to the management of oral habit-related malocclusions improves functional balance and quality of life during childhood, while reducing the complexity of comprehensive treatment. These principles represent the primary purpose of the “Orange July” campaign and MIO, which emphasizes timely preventive and interceptive approaches during childhood.

## RESUMO

**Introdução:**

Os hábitos bucais deletérios são frequentes durante a infância e, quando persistentes, podem influenciar o crescimento craniofacial e o desenvolvimento oclusal. Entre as más oclusões relacionadas aos hábitos bucais, podemos destacar a mordida aberta anterior (MAA) e a mordida cruzada posterior (MCP).

**Objetivos:**

Este artigo apresenta uma discussão conceitual e clínica sobre os hábitos bucais e suas repercussões no desenvolvimento da oclusão, com ênfase no diagnóstico precoce e na Ortodontia Minimamente Invasiva (OMI).

**Resultados:**

A literatura descreve associações entre sucção não nutritiva, interposição lingual, respiração bucal e alterações oclusais, especialmente a MAA dentoalveolar e a deficiência transversal da maxila. As abordagens interceptoras relatadas priorizam a interrupção dos hábitos, associada à indicação de dispositivos ortodônticos simples e ao manejo interdisciplinar.

**Conclusões:**

A abordagem ortodôntica interdisciplinar das más oclusões relacionadas aos hábitos bucais deletérios é capaz de melhorar o equilíbrio funcional e a qualidade de vida das crianças, ao mesmo tempo em que reduz a complexidade do tratamento ortodôntico corretivo. Esses princípios representam o principal propósito da campanha “Julho Laranja” e da OMI.

## INTRODUCTION

Human behavior is influenced by repetitive actions that, over time, may become automatic and incorporated into daily routines. These actions, often performed unconsciously, are referred to as habits^1^ and may exert relevant effects on individual health and development. When they involve the orofacial region, they are defined as oral habits, including practices such as digit sucking, pacifier or bottle use, tongue interposition, mouth breathing, and lip sucking, which may interfere with the growth and function of the stomatognathic system.^2^ ^,^ ^3^

The prevalence of oral habits in childhood varies widely across studies. During the primary dentition stage, approximately half of children present one or more habits.^4^ Although there is a tendency toward spontaneous reduction in early childhood, the persistence of these habits is frequent and influences craniofacial growth and occlusal development.^2^ ^,^ ^3^ ^,^ ^5^

The maintenance of oral habits is associated with the occurrence of malocclusions, particularly anterior open bite (AOB), posterior crossbite (PCB), and increased overjet.^6^ The severity of these changes depends on the duration, frequency, and intensity of the habit, as described by Graber.^7^ In this context, the American Academy of Pediatric Dentistry recommends early identification of deleterious habits and attention to their potential impact on the craniofacial complex.^8^

The American Association of Orthodontists recommends the first orthodontic evaluation by seven years of age. In Brazil, the “Orange July” campaign has contributed to raising awareness among general dentists and parents regarding early orthodontic evaluation at the beginning of the mixed dentition, aiming to promote timely interceptive treatments and disseminate the concept of Minimally Invasive Orthodontic (MIO) management, described in Part I of this article series.^9^ Within this context, this article provides a conceptual and clinical overview of oral habits and their impact on occlusal development, with an emphasis on early diagnosis and MIO.

## ORAL HABITS IN ORTHODONTICS

Among the most frequent oral habits in childhood are sucking habits, tongue interposition, and mouth breathing. Sucking habits may be classified as nutritive, such as breastfeeding and bottle feeding, or non-nutritive, mainly represented by pacifier use and digit sucking. Sucking is an innate mammalian behavior, present since intrauterine life, essential for survival in the first months of life and for stimulation of the orofacial musculature.^10^

Breastfeeding plays a fundamental role in children’s general health, providing nutritional, immunological, and psychological benefits, in addition to favoring adequate craniofacial growth.^11^ ^,^ ^12^ Sucking during breastfeeding promotes balance between internal and external oral musculature, contributing to normal occlusal development in the primary dentition.^13^ Evidence indicates that shorter breastfeeding duration is associated with higher prevalence of non-nutritive sucking habits and increased risk of malocclusion development.^14^ ^-^ ^16^ Moreover, breastfeeding between six and twelve months has a protective effect against mouth breathing.^17^

When breastfeeding is not possible, bottle feeding may be introduced. However, because it requires less muscular effort and orofacial coordination, it may favor the development of non-nutritive sucking habits, such as pacifier use or digit sucking.^3^ ^,^ ^18^ Unlike natural breastfeeding, artificial feeding does not provide adequate muscular stimulation, and parents should therefore be advised regarding the use of less harmful nipples.

From an orthodontic perspective, nutritive sucking habits are not considered deleterious, as they occur for limited periods. In contrast, non-nutritive sucking habits are more directly associated with the development of malocclusions and will be specifically addressed in the following sections.^7^

## NON-NUTRITIVE SUCKING HABITS: DIGIT SUCKING AND PROLONGED PACIFIER USE

Non-nutritive sucking habits (NNSH) correspond to sucking behaviors without a feeding purpose, such as pacifier use and digit sucking. Several theories explain their occurrence in childhood, and they are often associated with emotional aspects and tension relief, considering that sucking functions involving fingers, tongue, and lips are present from the prenatal period as instinctive mammalian behaviors.

Pacifier sucking is the most prevalent non-nutritive habit, with reported frequencies ranging from 13% to nearly 100%, depending on the population studied.^19^ As early as five months of age, approximately 82% of children may present NNSH.^20^ In most cases, spontaneous cessation of the oral habit occurs with emotional maturation, and the prevalence of non-nutritive sucking habits is estimated to decrease from 63% to 40% between 15 and 36 months of age^21^

When persistent, particularly beyond three years of age, NNSH are significantly associated with alterations in occlusal development.^2^ ^,^ ^3^ A systematic review demonstrated that these habits increase the likelihood of malocclusion development, with the severity of alterations being directly proportional to the duration of force application, and time accounting for approximately 50% of the observed impact.^16^

Pacifier design may also influence its effects on the stomatognathic system. In a computational simulation study, orthodontic pacifiers and standard models exhibited less dental displacement compared with conventional pacifiers, suggesting lower mechanical load on palatal tissues and a potential reduction in malocclusion risk.^22^

The main difference between pacifier use and digit sucking lies in their duration, as the latter tends to persist for longer periods.^23^ Given the difficulty in eliminating this habit, initial substitution of digit sucking by pacifier use may be considered a transitional strategy.

Additionally, co-sleeping, defined as bed-sharing or excessive proximity between mother and infant, lasting less than six months, has been associated with a higher prevalence of NNSH, including pacifier use and digit sucking, as well as with atypical swallowing, potentially related to higher levels of infant anxiety.^24^

## TONGUE INTERPOSITION: ANTERIOR TONGUE POSTURE AND INTERPOSITION DURING SWALLOWING, SPEECH, AND/OR AT REST

Tongue interposition, also referred to as atypical swallowing or persistent infantile swallowing pattern, is characterized by anterior projection of the tongue against or between the teeth during swallowing, speech, or at rest. When maintained, this continuous pressure may result in dental and functional alterations.

In the first months of life, an infantile swallowing pattern is expected, in which the tongue is positioned between the bony bases. With dental eruption, a transition to mature swallowing occurs, with the tongue tip positioned at the level of the retroincisal papilla, generally around 15 months of age.^25^ ^-^ ^28^ Failure in this process characterizes atypical swallowing, a condition associated with the development of malocclusions, especially AOB and PCB.

Atypical swallowing may be classified as primary or secondary. In the primary form, emotional factors may contribute to the persistence of the infantile pattern, particularly under stress. In the secondary form, external factors play a relevant role, including non-nutritive sucking habits, prolonged artificial feeding, adenoid and tonsillar hypertrophy, allergic conditions, and other functional alterations.^28^

Regardless of etiology, tongue interposition interferes with occlusal development through neuromuscular imbalance, favoring the establishment or maintenance of malocclusions. Therefore, its management often involves orofacial myofunctional therapy conducted by speech-language pathologists, combined with orthodontic intervention to restore proper tongue positioning.^28^ ^,^ ^29^

## MOUTH BREATHING AND ITS RELATIONSHIP WITH PEDIATRIC OBSTRUCTIVE SLEEP APNEA (OSA)

Under normal physiological conditions, breathing occurs predominantly through the nasal airway, allowing adequate warming, humidification, and filtration of inspired air. Mouth breathing manifests when obstructions are present in the upper airways, located in the nasal cavity (turbinate hypertrophy, septal deviation, allergic rhinitis), nasopharynx (adenoid hypertrophy), or oropharynx (palatine tonsil hypertrophy).

Mouth breathing is associated with postural adaptations, such as parted lips, inferior tongue positioning, and posterior mandibular rotation, favoring increased lower anterior facial height (LAFH) and the development of AOB.^30^ Tongue anteriorization and inferiorization, due to the lack of palatal contact, contribute to maxillary constriction and PCB development, while posterior mandibular rotation acts as a compensatory mechanism through increased anterior alveolar development. When this compensation is insufficient, AOB becomes clinically evident, accentuating the vertical growth pattern in mouth-breathing children. However, not all vertical imbalance is associated with mouth breathing, as approximately 70% of dolichofacial patients exhibit predominantly nasal breathing.^31^

The orthodontist plays a fundamental role in identifying this breathing pattern. Anamnestic findings such as restless sleep, snoring, excessive drooling on the pillow, and fatigue during physical activity, combined with clinical signs (long and narrow face, predominance of the lower facial third, absence of passive lip seal, hypotonic lips, narrow nostrils, and dark circles) and occlusal features (AOB and maxillary constriction, with or without PCB), may suggest mouth breathing. Radiographic findings compatible with adenoid and tonsillar hypertrophy complement the diagnosis, and referral to an otolaryngologist is indicated for definitive evaluation.

It should be emphasized that signs commonly attributed to mouth breathing are also observed in children with pediatric OSA. Approximately 10% of orthodontic patients present suggestive signs of OSA, compared with about 4% of the general pediatric population,^32^ supporting the recommendation for the use of screening instruments such as the Pediatric Sleep Questionnaire.^33^

Pediatric OSA is characterized by recurrent episodes of partial or complete upper airway obstruction during sleep, associated with oxygen desaturation and micro-arousals,^34^ ^,^ ^35^ representing a significant public health problem.^36^ As it occurs during a critical period of neural maturation, OSA is associated with neurocognitive impairments, including deficits in attention, memory, and academic performance, as well as increased risk of behavioral alterations, including symptoms consistent with attention-deficit/hyperactivity disorder (ADHD), compared with children without the disorder.^37^ ^-^ ^39^ Early diagnosis and referral are essential to minimize its impact on growth and craniofacial development.

In the anamnesis and clinical examination of pediatric patients, particularly in children with signs suggestive of sleep-disordered breathing, sleep bruxism (SB) should also be investigated as its association with pediatric OSA has been considered plausible.^40^ Although malocclusion is not considered a direct cause or consequence of SB, excessive dental wear and symptoms of temporomandibular joint tenderness and masticatory muscle pain may be present and should be taken into account when referring the child to a temporomandibular disorder and orofacial pain specialist.

## MALOCCLUSIONS RELATED TO ORAL HABITS AND INTERCEPTIVE ORTHODONTIC MANAGEMENT

Persistent deleterious oral habits may disrupt the balance of intraoral and perioral forces responsible for normal facial and dental development.^2^ ^,^ ^3^ When maintained beyond the initial phase of primary dentition, these habits predominantly affect the vertical and transverse aspects of the craniofacial complex, often associated with dysfunctional orofacial patterns. Among 2,016 children with non-nutritive sucking habits, only 20% presented no malocclusion.^5^ AOB was the most prevalent alteration, affecting approximately 50% of children in the primary dentition, followed by PCB (18%) and the association of both (10%), as illustrated in Figure 1.^5^ Aligned with the principles of MIO, the following section addresses interceptive strategies for correcting AOB and PCB, highlighting the importance of early diagnosis and interdisciplinary care in growing patients.

**Figure 1:**
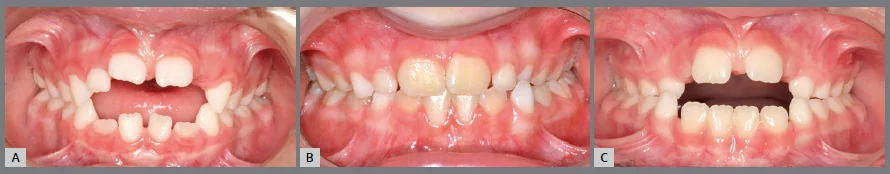
Malocclusions associated with oral habits in the mixed dentition: **A**) anterior open bite (AOB); **B**) posterior crossbite (PCB); (**C**) association between AOB and PCB.

## ANTERIOR OPEN BITE

Anterior open bite (AOB) is defined as the absence of contact between the maxillary and mandibular incisors when posterior teeth are in occlusion.^41^ ^,^ ^42^ Its etiology is multifactorial, involving interactions between genetic and environmental factors.^43^ When environmental contribution predominates, prognosis and stability tend to be favorable, as the etiological factor can be eliminated, as observed in dentoalveolar AOB associated with persistent oral habits. In the presence of habits, tongue displacement leads to muscular and functional imbalance (Fig. 2). Additionally, habits such as thumb sucking act as physical barriers, interfering with anterior dental development, resulting in the AOB (Fig. 1).

**Figure 2:**
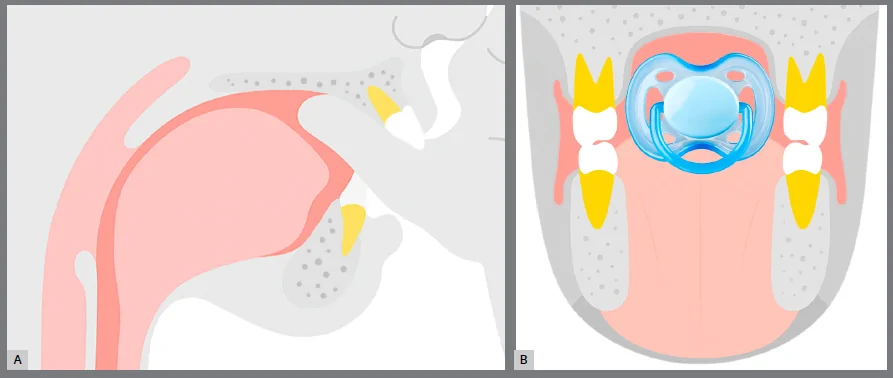
Muscular imbalance in the presence of deleterious oral habits. Digit sucking results in AOB, PCB, and increased overjet, acting as a physical barrier to anterior tooth eruption and promoting dental tipping (**A**). Subsequently, predominance of external musculature associated with inferior tongue positioning favors maxillary constriction and PCB (**B**).

The prevalence of AOB decreases from primary to mixed dentition.^44^ ^,^ ^45^ In the primary dentition, its frequency is approximately 27%, reaching up to 50% in the presence of sucking habits, whereas in the mixed dentition it decreases to around 17%. This reduction is related to the high likelihood of spontaneous correction following habit interruption in childhood,^46^ as illustrated in Figure 3.

**Figure 3:**
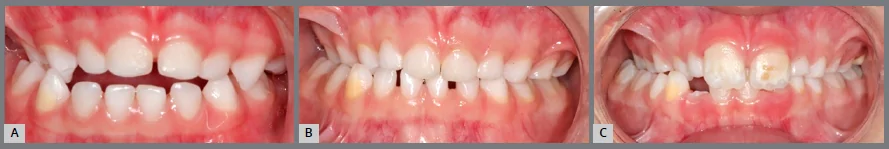
Two-year-old patient with AOB and PCB associated with pacifier use (**A**). Spontaneous correction of AOB after habit interruption was observed (**B**), while PCB persisted until the mixed dentition (**C**).

When spontaneous correction does not occur, early orthodontic intervention may be indicated from six years of age or from the mixed dentition stage. This approach is supported by the negative impact of AOB on children’s quality of life,^47^ improvement following interceptive orthodontic treatment,^48^ ^,^ ^49^ and stability of outcomes,^50^ ^,^ ^51^ justifying management of this malocclusion in childhood.

Among therapeutic options, the palatal crib stands out as one of the most commonly used and effective devices. The original design proposed in 1950^52^ has been largely preserved and acts as a physical obstacle to habits, allowing passive correction of the malocclusion. Among available variations, the fixed palatal crib (Fig. 4) is preferred, due to reduced dependence on patient compliance. Studies have demonstrated 90% effectiveness of fixed cribs in correcting AOB in the mixed dentition, compared with 60% for removable alternatives.^53^

**Figure 4:**
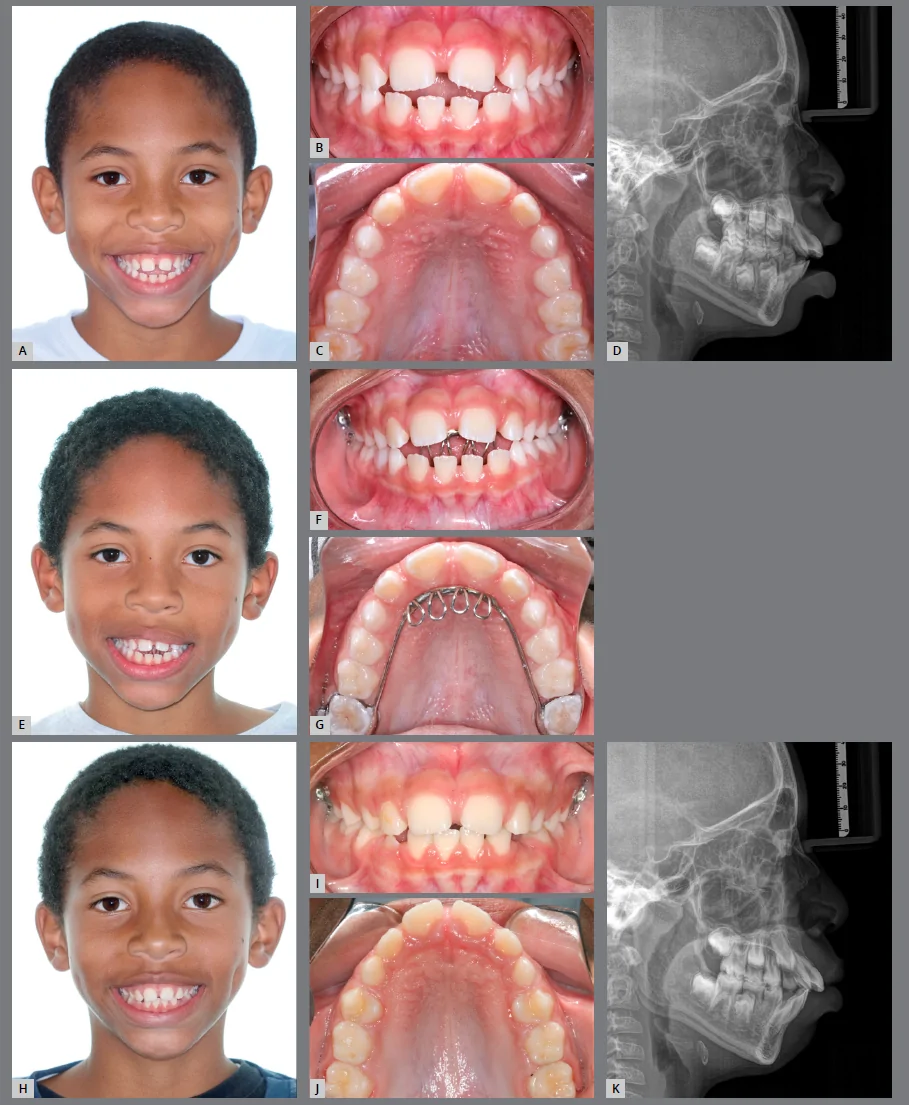
A-D) Eight-year-old patient with AOB. E-G) Installation of fixed palatal crib, and H-K) passive correction of AOB through dentoalveolar effects.

Bonded lingual spurs represent an additional alternative for interceptive treatment of AOB (Fig. 5), offering low cost and good esthetics due to placement on the lingual surfaces of incisors.^54^ However, they present lower effectiveness in habit elimination, compared with fixed palatal cribs, as well as higher rates of fractures and clinical complications.^55^ ^,^ ^56^

**Figure 5:**
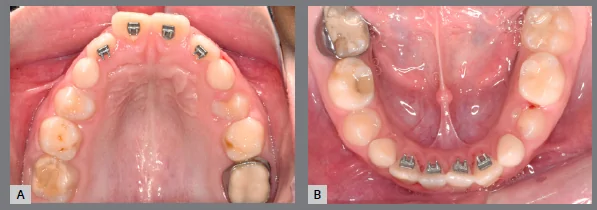
Bonded lingual spurs on the lingual surfaces of maxillary (**A**) and mandibular (**B**) incisors for AOB correction in the late mixed dentition.

Recently, the esthetic palatal crib was described (Fig. 6), making the device more discreet in the smile.^57^ Its esthetic appeal may enhance treatment acceptance without compromising effectiveness.

**Figure 6:**
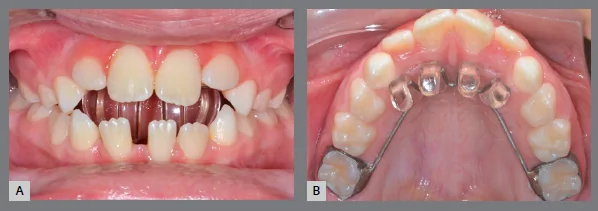
Esthetic palatal crib for AOB correction: frontal (**A**) and occlusal (**B**) views.

Classic early interventions for AOB are characterized by simple, low-cost approaches in which orthodontic devices assist in controlling oral habits, favoring their interruption and appropriate occlusion development. This management allows early and passive correction of vertical overlap and, combined with widely documented clinical stability, remains the gold standard for treating dentoalveolar AOB in childhood.

## POSTERIOR CROSSBITE

The posterior crossbite (PCB) is characterized by a transverse discrepancy between the maxillary and mandibular dental arches,^58^ and may present unilaterally or bilaterally (Fig. 1B). Spontaneous correction of this discrepancy is uncommon.^59^ Epidemiological studies have reported a PCB prevalence of 20% in the primary dentition^5^ and 18% in the mixed dentition,^60^ reinforcing its limited self-correction potential. Higher prevalence is observed in girls and children attending public schools, possibly related to higher frequency of sucking habits.^5^

Persistent oral habits displace the tongue inferiorly, reducing internal transverse force in the maxilla. Consequently, external musculature, particularly the buccinator muscle, becomes predominant, favoring maxillary constriction and PCB (Fig. 2).

Resolution of transverse discrepancies is considered a priority in orthodontic planning and is recommended from five years of age. Several appliances may be used for PCB correction, and clinical selection should be aligned with scientific evidence regarding the effects and indications of each option. Rapid maxillary expansion (RME) has been the treatment of choice, given its clinical effectiveness and predictability.^61^ ^,^ ^62^ Figure 3 illustrates a two-year-old patient with AOB, PCB, and pacifier use. After habit interruption, spontaneous correction of AOB occurred; however, PCB persisted until the mixed dentition, and was treated with RME using a Hyrax expander (Fig. 7).

**Figure 7:**
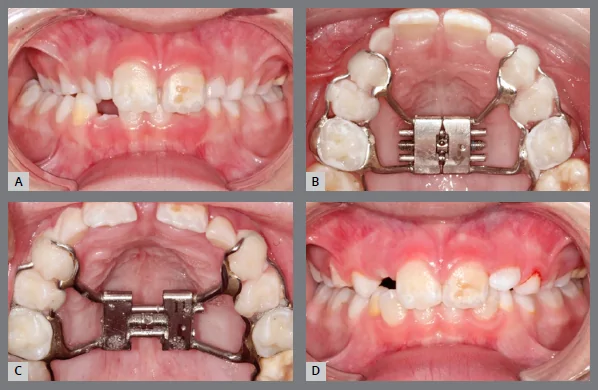
Treatment of PCB in the patient from Figure 3: **A**) Initial posterior crossbite, **B**) rapid maxillary expansion with a Hyrax expander, **C**) interincisal diastema after screw activation and **D**) transverse correction after appliance removal.

The treatment of PCB is recommended during the mixed dentition due to its minimal potential for spontaneous correction and the documented stability of the outcomes,^5^ ^,^ ^59^ ^,^ ^63^ Additionally, the increased skeletal bioplasticity and the lower degree of midpalatal suture interdigitation during this developmental stage facilitate transverse maxillary correction.^58^ Finally, early correction of PCB contributes to proper condylar positioning within the glenoid fossa, as well as to favorable growth, development, and correct relationships of the skeletal bases and dental arches.^64^ ^,^ ^65^

The association between PCB and AOB occurs in approximately 10% of children in the primary dentition.^5^ In such cases, treatment should prioritize correction of the transverse discrepancy. In many patients, especially when maxillary expansion coincides with habit interruption, spontaneous correction of AOB is observed. Otherwise, a fixed palatal crib may be used as retention, after expander removal, or incorporated into the expander itself, provided it is fabricated in two segments to allow screw activation.

Clinical and epidemiological data reinforce the importance of early diagnosis and timely intervention. Although orthodontic correction is essential for restoring maxillary width, stability of results often depends on management of associated functional factors, justifying an interdisciplinary approach, discussed below.

## INTERDISCIPLINARY MANAGEMENT OF ORAL HABIT-RELATED MALOCCLUSIONS

Correction of AOB and PCB contributes to improved smile esthetics and balance of the stomatognathic system. However, interception of oral habits and associated functional alterations often requires a coordinated interdisciplinary approach involving orthodontists, pediatric dentists, physicians, speech-language pathologists, and, in some cases, psychologists.

In pediatric patients, breathing patterns should be carefully evaluated. Investigation includes anamnesis, clinical examination of palatine tonsils, airway assessment through radiographic exams, and use of validated screening instruments such as the Pediatric Sleep Questionnaire.^66^ In the presence of signs or symptoms of predominantly oral breathing, referral to a pediatric otolaryngologist is recommended before initiating orthodontic intervention.^67^

Interceptive orthodontic treatment may promote functional reorganization of the orofacial system, contributing to proper tongue positioning, maturation of swallowing patterns, and reduction of speech distortions. Normalization of tongue function may occur spontaneously after morphological correction and habit interruption or, when necessary, be enhanced through orofacial myofunctional therapy conducted by a speech-language pathologist before, during, or after correction of AOB or PCB.

Management of oral habits in children should consider emotional and behavioral aspects, as treatment adherence largely depends on the child’s readiness and family involvement. Behavioral guidance and, when indicated, psychological support may be decisive for habit cessation and result stability.

By integrating airway evaluation, morphological correction, and functional rehabilitation, an interdisciplinary approach contributes to more comprehensive and stable outcomes in the treatment of oral habit-related malocclusions.

## FINAL CONSIDERATIONS

Oral habits and the associated malocclusions exert a significant influence on craniofacial growth. In this context, the “Orange July” campaign and the concept of MIO reinforce the importance of timely preventive and interceptive interventions during childhood. From an orthodontic perspective, AOB and PCB represent the main malocclusions related to deleterious oral habits. Orthodontic and interdisciplinary management of these conditions promotes functional balance and improves children’s self-esteem and quality of life. Moreover, it contributes to reducing the complexity of comprehensive orthodontic treatment, thereby fulfilling the fundamental principles of preventive and interceptive orthodontics.

## Data Availability

Data Availability Statement: All data generated or analyzed during this study are included in this published article.
